# Cognitive Aging in the United States and Mexico

**DOI:** 10.1093/geroni/igaa057.1903

**Published:** 2020-12-16

**Authors:** Sunshine Rote, Jacqueline Angel, Fernando Torres-Gil

## Abstract

The Latino population is rapidly aging, with the number of adults 65 and older expected to increase by more than six times to 17.5 million by 2050. Mexico’s population is also aging and will increase by 227 percent over the next 25 years. We focus on the consequences of rising longevity and increasing numbers of older Latinos living with dementia both in the U.S. and in Mexico. Providing cost-effective and appropriate services to aging Latinos with dementia will require a clear understanding of the intra-diversity among this group in different social and national circumstances. The purpose of this symposium is the understand how migration between and within countries and other social and health factors (e.g., diabetes) impact risk for cognitive impairment and dementia using three national datasets: the HRS, MHAS, and HEPESE. Four paper presentations and one discussant will examine several thematic issues as they relate to cognitive aging for Latinos, including: (1) cross-national estimates of dementia prevalence in Mexico and the U.S.; (2) the healthy immigrant effect and health convergence hypothesis for cognitive impairment for Latinos in the U.S. and Mexico; and (3) implications of these trends for long-term care service needs for Latinos living with dementia in the U.S. and Mexico. The resulting discussion will provide new empirical and theoretical insights on the determinants of cognitive aging for this population. It will also inform debates and aid in implementing innovative strategies and solutions to mitigate risk for impairment and improve dementia care for older Latinos.

